# P-1919. Universal Decolonization Reduced Antibiotic Use in Nursing Homes Participating in the SHIELD Regional Collaborative

**DOI:** 10.1093/ofid/ofaf695.2088

**Published:** 2026-01-11

**Authors:** Seong Eun Kim, Gabrielle Gussin, Ken Kleinman, Thomas T Tjoa, Raveena Signh, Raheeb Saavedra, Loren G Miller, Susan Huang

**Affiliations:** Chonnam National University Medical School, GwangJu, Kwangju-jikhalsi, Republic of Korea; University of California, Irvine School of Medicine, Division of Infectious Diseases, Irvine, California; University of Massachusetts Amherst, Amhurst, Massachusetts; University of California, Irvine School of Medicine, Division of Infectious Diseases, Irvine, California; University of California Irvine, Irvine, California; University of California, Irvine School of Medicine, Division of Infectious Diseases, Irvine, California; Lundquist Institute at Harbor-UCLA Medical Center, Los Angeles, CA; University of California, Irvine School of Medicine, Irvine, California

## Abstract

**Background:**

The SHIELD Regional Collaborative (JAMA 2024, PMID: 38557703) was a quasi-experimental decolonization initiative that found a 27% reduction in infection-related hospitalizations among the 16 nursing homes that adopted universal decolonization— chlorhexidine bathing and nasal iodophor. We evaluated whether this intervention also reduced antibiotic use.

**Methods:**

This SHIELD secondary analysis compared 16 participating nursing homes vs 45 non-participants in Orange County, CA to evaluate the effect of universal decolonization on antibiotic use in residents, defined as the number of days of antibiotic administration during the 7 days reported for each Minimum Data Set (MDS) assessment (admission, quarterly, discharge). We conducted a difference-in-differences analysis using generalized linear mixed logistic regression models to account for clustering within facility, and to assess the relative change in the odds of antibiotic use using an interaction term between participation status and time period.

**Results:**

Compared with the baseline period, decolonization nursing homes had a 48.1% reduction (OR=0.519, 95% CI: 0.512-0.527) in antibiotic use in the intervention period compared to a 41.2% reduction in routine care nursing homes (OR=0.588, 95% CI: 0.577-0.599), resulting in an 11.7% greater reduction in the odds of antibiotic use (relative OR=0.883; 95% CI 0.854-0.912; p< 0.0001) in decolonization nursing homes after the intervention, adjusting for age, gender, race, ethnicity, insurance, and coexisting conditions. Predicted probabilities showed antibiotic use decreased from 6.9% to 3.7% in the decolonization group compared to a decline from 7.0% to 4.2% in the routine care group.

**Conclusion:**

These findings support universal decolonization as an effective strategy to reduce antibiotic use in nursing homes. In addition to improved health outcomes, this reduction in antibiotic use plus the previously shown reduction in infection-related hospitalizations supports universal decolonization as a cost-saving strategy for nursing homes.

**Disclosures:**

Loren G. Miller, MD MPH, Armata: Grant/Research Support|GSK: Grant/Research Support|Merck: Grant/Research Support|Paratek: Grant/Research Support Susan Huang, MD, MPH, Xttrium: Conducting studies in which participating nursing homes and hospitalized patients receive contributed antiseptic products|Xttrium Laboratories: Conducting studies in which participating nursing homes and hospitalized patients receive contributed antiseptic product

